# E-Cigarette Use among Current Smokers Experiencing Homelessness

**DOI:** 10.3390/ijerph18073691

**Published:** 2021-04-01

**Authors:** Arturo Durazo, Marlena Hartman-Filson, Holly Elser, Natalie M. Alizaga, Maya Vijayaraghavan

**Affiliations:** 1 Center for Tobacco Control Research and Education, University of California, 530 Parnassus Avenue Suite 366, San Francisco, CA 94143, USA; Arturo.Durazo@ucsf.edu; 2 Division of General Internal Medicine, University of California, 1001 Potrero Avenue, San Francisco, CA 94110, USA; Marlena.Hartman-Filson@ucsf.edu; 3 Division of Adolescent Medicine, Department of Pediatrics, Stanford University, 770 Welch Road, Suite 100, Palo Alto, CA 94304, USA; holly.stewart@berkeley.edu; 4 Humanities and Social Sciences Division, Department of Psychology, Cañada College, 4200 Farm Hill Blvd, Bldg 03-139, Redwood City, CA 94061, USA; alizaganatalie@smccd.edu

**Keywords:** tobacco, homelessness, cigarettes, e-cigarettes, smoking cessation

## Abstract

Two-thirds of cigarette smokers experiencing homelessness report using alternative tobacco products, including blunts, cigarillos (little cigars) or roll-your-own tobacco or electronic nicotine delivery systems such as e-cigarettes. We examined attitudes toward e-cigarette use and explored whether e-cigarette use patterns were associated with past-year cigarette quit attempts among current smokers experiencing homelessness. Among the 470 current cigarette smokers recruited from homeless service sites in San Francisco, 22.1% (*n* = 65) reported the use of e-cigarettes in the past 30 days (‘dual users’). Compared to cigarette-only smokers, dual users considered e-cigarettes to be safer than cigarettes. Patterns of e-cigarette use, including the number of times used per day, duration of use during the day, manner of use and nicotine concentration were not associated with past-year cigarette quit attempts. Studies that examine the motivations for use of e-cigarettes, particularly for their use as smoking cessation aids, could inform interventions for tobacco use among people experiencing homelessness.

## 1. Introduction

Approximately 3.5 million individuals experience homelessness yearly in the United States. In San Francisco in 2019, over 8000 people had experienced homelessness, of whom 36% were sheltered and 64% were unsheltered. Black and Latinx populations are over-represented among populations experiencing homelessness, comprising 55% of the homeless community compared to 25% of the general population in San Francisco.

While rates of tobacco use have declined substantially in the general population, populations experiencing homelessness bear a disproportionate burden from tobacco use. In the United States (US), the prevalence of tobacco use among people experiencing homelessness (PEH) is estimated between 70% and 80% [1,2,3] compared to 13% in the general population [4,5]. 

As the landscape of available tobacco products has evolved to include alternative tobacco and nicotine products, the patterns of tobacco use have also changed among PEH. Two-thirds of current smokers who experience homelessness report the use of alternative tobacco products, including blunts and cigarillos (little cigars) or electronic nicotine delivery systems, with e-cigarettes being the most common form [6,7,8,9]. The concurrent use of these products and cigarette smoking might suggest increased nicotine dependence; however, few studies have explored this among PEH. 

Studies in the general population in the US have shown that people self-report using e-cigarettes to stop smoking, to reduce stress and to lose weight [10,11,12,13,14]. Several studies among PEH in the US have shown that e-cigarettes may be used as a smoking cessation aid, to circumvent indoor smoke-free policies or for the novelty and/or flavors of these products [3,6,7,8]. While some PEH may use e-cigarettes as an alternative, less expensive replacement to cigarettes, most PEH rely on little cigars or roll-your-own tobacco as the cheaper alternative to cigarettes [3,6,7,8]. In a study of smokers experiencing homelessness in Boston, approximately 60% who had used e-cigarettes in the past month did so because of reduced costs compared to cigarettes [6]. These data suggest that motivations for the use of e-cigarettes, including for smoking cessation, may differ based on affordability and accessibility to e-cigarettes [15,16]. 

Cigarette smokers experiencing homelessness are motivated to quit smoking and make quit attempts at similar rates compared to the general population, but their rates of successful quitting are low (quit ratio 9–13% compared to 50–60% in the general population) [17,18,19]. Use of approved cessation aids such as nicotine replacement therapy is low among PEH [20], which, in part, may contribute to the low rates of successful quitting. However, the low use of approved cessation aids among some smokers experiencing homelessness may also highlight a preference for electronic nicotine delivery devices such as e-cigarettes for smoking cessation [16]. 

While expert consensus in the US highlights the potential for reduced harm from e-cigarettes compared to cigarettes, it is currently not approved by the Food and Drug Administration for smoking cessation treatment [21]. In contrast, countries such as the United Kingdom (UK) have promoted the use of e-cigarettes as cessation aids, and recent trials highlight a potential role for e-cigarettes in smoking cessation among PEH [22,23]. In the UK, a cluster feasibility study of an intervention that provided e-cigarettes for smoking cessation to PEH showed an increased abstinence at 6 months in the intervention group (9% intervention compared to 0% in control) [24]. An uncontrolled trial of a small-scale intervention of e-cigarettes for smoking cessation showed improvements in self-reported reduction in consumption at a 12-weeks follow-up, but not in expired carbon monoxide among PEH in Ireland [25]. In the US, a single arm trial of an intervention that included e-cigarettes among cigarette smokers who had experienced homelessness showed an 18% increased odds of being abstinent for each week enrolled in the study [26]. However, these trials are of a small-scale and more data on the patterns of e-cigarette use could inform interventions that use e-cigarettes for smoking cessation among PEH. 

Moreover, attitudes toward e-cigarette use, including the perceived safety and cost of e-cigarettes could motivate the use of e-cigarettes [27,28]. In studies of adult smokers in the US, those who considered e-cigarettes as a safer alternative to cigarettes and that e-cigarettes helped people quit smoking, were more likely to use e-cigarettes [29,30,31]. However, studies also showed that positive perceptions about e-cigarette safety are associated with concurrent cigarette smoking and e-cigarette use among low-income racial/ethnic minorities and pregnant women [32,33], which may hinder cessation efforts. 

In this cross-sectional study, we explored attitudes toward e-cigarette use, the patterns of e-cigarette use and their associations with past-year cigarette quit attempts among the PEH in San Francisco. Because there may be a subset of PEH who are using e-cigarettes for smoking cessation, we examined whether specific patterns of e-cigarette use may be associated with cigarette quit attempts. We hypothesized that a more frequent use of e-cigarettes may be associated with quit attempts in the past year.

## 2. Materials and Methods

We conducted a cross-sectional study of PEH who were recruited from eight study sites, including emergency shelters, navigation centers, day-time referral programs and community centers serving adults experiencing homelessness in San Francisco, California [34,35]. Individuals were eligible to participate if they were 18 years and older, had smoked at least 100 cigarettes in their lifetime, currently smoked cigarettes defined as having smoked in the past 30 days, were receiving services at the recruitment site and were currently homeless as defined by the Homeless Emergency Assistance and Rapid Transition to Housing Act (e.g., unsheltered, in a temporary shelter or living doubled up with family or friends) [36]. 

### 2.1. Procedures

We recruited participants between November 2017 and July 2018 using a typical case sampling, a purposive sampling method [37,38,39], representing the average or typical smoker experiencing homelessness at these centers. We chose recruitment settings that focused exclusively on providing services to adults experiencing homelessness, including sheltered and unsheltered populations. The individuals receiving shelter and/or services at these sites were representative of the clientele who would reflect typical perspectives of current cigarette smokers with lived experiences of homelessness. Study staff recruited participants from common spaces in the recruitment sites where most people gathered. Service staff at the study sites helped identify participants who were representative of the average smoker experiencing homelessness. For each of the eight study sites, study staff chose random days and time periods for the recruitment. During recruitment times, study staff recruited participants by making announcements at the study sites in common areas and during community meetings and by posting flyers. During the selected dates and times for recruitment, study staff would approach potential participants, screen them and enroll eligible participants into the study. We did not gather information on the total number of participants approached at each session. On each recruitment day, study staff reviewed the list of individuals screened and recruited to ensure that there were no duplicate records. On instances where there were duplicates, we retained only the first record for that participant. Study staff administered a 20 min questionnaire on a tablet device in a private room at the study site. Participants who completed the questionnaire received a $15 gift card for their participation, an amount that we have found to be sufficient to reimburse participants for their time without a risk of coercion. This type of reimbursement was similar to what we have used in previous studies among PEH [40,41]. All study procedures were approved by the University of California, San Francisco Committee on Human Research.

### 2.2. Sociodemographic Characteristics

Participants self-reported their age, gender (female, male or transgender) and race and ethnicity (African–American/Black, American Indian/Alaska Native, Asian/Pacific Islander, Hispanic/Latinx, White or more than one ethnic group). 

### 2.3. Cigarette Smoking Behaviors

We classified current cigarette smokers as participants who reported smoking a cigarette in the past 30 days. We asked participants to report the average number of cigarettes smoked per day and the time to their first cigarette after waking (within 5 min, 6–30 min, 31–60 min or after 60 min). We asked participants to report whether they had attempted a quit for one day or longer in the past year. 

### 2.4. Patterns of E-Cigarette Use

We classified dual users as participants who reported the concurrent use of e-cigarettes in the past 30 days with current cigarette smoking and the remaining sample as cigarette-only users. For participants who reported the current use of e-cigarettes (i.e., use in the past 30 days), we asked how many times per day they had used their e-cigarettes (assuming that one ‘time’ consisted of 15 puffs or lasted around 10 min) [42]. Participants reported the duration of time that they had used their e-cigarettes on a given day (just a few puffs (less than 1 min total), 1–5 min total, 6–15 min total, 16–60 min total, more than 1 h of total use) and the manner in which they had used their e-cigarettes (continuously throughout the day, distinct smoking bouts that are shorter than smoking a traditional cigarette, distinct smoking bouts similar to smoking a traditional cigarette or other). We asked participants to report the concentration of nicotine usually used in their e-cigarettes (don’t know, 0 mg, 1–6 mg, 7–12 mg, 13–18 mg, 19–24 mg or 25+ mg). 

### 2.5. Attitudes toward E-Cigarettes

We asked participants to indicate their level of disagreement or agreement with statements about cigarettes and e-cigarettes. Participants responded to statements about cigarette smoking cessation (e.g., “people who smoke cigarettes should quit”), use of tobacco or e-cigarettes (e.g., “e-cigarettes should be allowed in outdoor spaces”), perceptions of cigarette or e-cigarette harm and safety (e.g., “e-cigarettes are safer than smoking”) and nicotine and tar content (e.g., “e-cigarettes don’t contain tar”). We dichotomized responses as ‘strongly disagree/disagree/neutral’ versus ‘agree/strongly agree’.

### 2.6. Source of E-Cigarettes

For participants who reported the current use of e-cigarettes (i.e., use in the past 30 days), we asked them to report where they had obtained their e-cigarettes during their most recent purchase (i.e., liquor store, convenience store or gas station, supermarket or drug store, smoke shop, vape shop or marijuana dispensary or friends or strangers).

### 2.7. Data Analysis

We calculated the means and standard deviations (SD) for the continuous variables and proportions for the categorical variables by tobacco use status. We reported the differences in demographics, cigarette smoking and/or e-cigarette use characteristics and attitudes toward cigarettes and e-cigarettes use between cigarette-only smokers and dual users. We examined the bivariate association between e-cigarette use patterns and past-year cigarette quit attempts. Using logistic regression, we examined bivariate and multivariable associations between attitudes toward e-cigarette use and cigarette quit attempts. We adjusted for covariates shown to be associated with cigarette quit attempts in prior studies among PEH, including demographics (age, gender and race/ethnicity) and nicotine dependence measures (average number of cigarettes per day and time to first cigarette after waking) [2,20,35]. We conducted all analyses using Stata, Version 15.1 (StataCorp LLC, College Station, TX, USA).

## 3. Results

Among the 470 participants, the average age was 49.9 years (SD = 11.6), the majority were male (66.2%) and of African–American/Black race/ethnicity (44.5%) (Table 1). Participants reported an average of 11.3 cigarettes smoked per day (SD = 10.5) and 44.3% had made a quit attempt in the past year. Out of the current smokers, 86.2% were cigarette-only smokers only and 13.8% were dual users (*n* = 65). Dual users were younger than cigarette-only smokers. Cigarette-only smokers were represented more among African–American participants, whereas dual users were represented more among Latinx, multi-racial and non-Hispanic White participants. Among the participants, 43.2% of cigarette-only smokers and 50.8% of dual users had made a quit attempt in the past year. A higher proportion of dual users were likely to report smoking within 30 min of waking (Table 1). The most common sources of e-cigarettes were a smoke/vape shop, a marijuana dispensary or friends or strangers.

Participants who had made a quit attempt in the past year reported having used their e-cigarettes more times during the day (6.4 times versus 4.8 times) and for a longer duration of time than those who had not made a quit attempt (Table 2). Similarly, those who had made a quit attempt appeared to use their e-cigarette throughout the day continuously compared to those who had not. Knowledge of the concentration of e-cigarettes did not appear to differ by quit attempts; however, most participants reported not knowing the concentration of nicotine in their e-cigarette products.

The majority of participants agreed that cigarettes and e-cigarettes were harmful to children and that these products should be used outdoors. Fewer than 25% of participants agreed that cigarettes should be used indoors, whereas more than 30% agreed that e-cigarettes should be permitted for indoor use. About half the sample agreed that e-cigarettes help people quit cigarette smoking. Dual users (49.2%) were more likely to consider e-cigarettes as safer than cigarettes compared to cigarette-only smokers (36.1%) (Table 3).

In bivariate analyses, nicotine dependence variables, including a higher cigarette consumption and time to first cigarette after waking within 30 min, were associated with lower odds of a quit attempt in the past year (Table 4). In the adjusted model, cigarette smokers who agreed that smokers should quit were more likely to make a quit attempt in the past year (aOR = 2.15; 95% CI 1.22, 3.79), whereas those who had smoked within 30 min from waking up were less likely to make a quit attempt in the past year (aOR = 0.57; 95% CI 0.33, 0.96). Current e-cigarette use was not associated with a quit attempt in the past year.

## 4. Discussion

In this cross-sectional study among PEH, we found that 13.8% of participants were dual users of cigarettes and e-cigarettes, a proportion higher than the general population (1.4%) [43]. Dual use was not associated with past-year cigarette quit attempts. Among dual users, the patterns of e-cigarette use—including the number of times used per day, the duration of time used per day, the manner in which they were used and the knowledge of the nicotine concentration—were not associated with past-year cigarette quit attempts. 

Recent studies have suggested that the patterns of e-cigarette use may signify cigarette-smoking reduction or cessation behaviors [44]. In a national longitudinal study in the US of cigarette smokers who were 25 years or older, those who initiated the daily use of e-cigarettes between baseline and one year follow-up were more likely to have quit smoking or show a reduced consumption compared to those who had not initiated e-cigarettes [45]. Moreover, those who were using e-cigarettes less frequently did not have higher cessation attempts or show a reduction in consumption, suggesting that the frequency of e-cigarettes use may be an important factor for smoking cessation. An uncontrolled trial of an intervention that included the provision of daily e-cigarettes to long-term smokers with serious mental illness (*N* = 21) showed a reduction in cigarette consumption and exhaled carbon monoxide between baseline and 4-weeks follow-up among study participants. These findings suggest a potential role for e-cigarettes as a substitute for cigarettes [46]. 

In our study, a slightly higher number of dual users who attempted to quit smoking in the past year used e-cigarettes more times during the day, throughout the day (34.4%), or greater than 1 h at one sitting (12.5%) compared to those who did not attempt to quit. While we may be underpowered to detect associations between these patterns of e-cigarette use and past-year cigarette quit attempts, our study highlights a sub-sample of smokers who may be using e-cigarettes for smoking cessation. Studies that compare users who have switched completely to e-cigarettes versus those who are dual users may provide insights into the role of e-cigarettes in smoking cessation among PEH.

Over half of the participants reported not knowing the nicotine concentration in their e-cigarettes. Most studies that explored e-cigarette use among PEH have focused on their motivations of use, the effects of e-cigarettes on smoking cessation or subjective experiences of use of e-cigarettes [8,25,26]. It is unknown whether the knowledge of nicotine concentration could be associated with the use of e-cigarettes for smoking cessation among PEH and this relationship warrants further exploration. 

Over two thirds of cigarette-only smokers and dual users agreed that people who smoke should quit smoking. About half the sample agreed that e-cigarettes could be used as a cessation aid, consistent with prior studies among PEH who showed a preference for alternative forms of nicotine delivery for smoking cessation [16]. Fewer participants agreed that cigarettes should be used indoors, highlighting that social norms around second-hand smoke risk were prevalent among participants. Yet, 40% agreed that the indoor use of e-cigarettes should be allowed, highlighting a role for education around the potential harms of the indoor exposure to e-cigarette aerosol [47]. 

Our study has limitations. The data on tobacco use were self-reported and not biochemically verified, which may have led to a misclassification bias. The cross-sectional approach does not allow for a causal inference. This study may not be generalizable to populations of homeless adults from other regions in the US. The small sample size of dual users allowed us to conduct exploratory analyses of patterns of e-cigarette use and their association with quit attempts that need to be confirmed in larger, well-powered studies. Our study was conducted during an ongoing public health campaign to ban flavored tobacco and nicotine products [48]. While this may have influenced attitudes toward these products, the direction of these influences is unclear.

## 5. Conclusions

Despite these limitations, our study extends findings from previous studies by highlighting specific patterns of e-cigarette use among PEH that may have implications for smoking cessation. PEH have among the highest rates of tobacco use and the lowest rates of successful smoking cessation. While promoting the consistent use of guideline-based cessation aids is the primary treatment for smoking cessation, there may be a role for alternative treatments among smokers with previous unsuccessful cessation attempts. Our study highlights a sub-sample of smokers experiencing homelessness who may prefer e-cigarettes for smoking cessation. Future studies, including observational studies and randomized controlled trials, are needed to determine the efficacy and the safety of e-cigarettes for smoking cessation treatment among people experiencing homelessness.

## Figures and Tables

**Table 1:** Sample characteristics and tobacco use among cigarette-only smoking and dual users (cigarette and e-cigarette use) in the past 30 days.

Variable	Overall (*n* = 470)	Cigarette-Only (*n* = 405)	Dual Users (*n* = 65)
	*n* (%)	*n* (%)	*n* (%)
Age—mean (SD)	49.9 (11.6)	50.5 (11.6)	45.8 (10.6)
Gender			
Female	145 (31.1)	121 (30.1)	24 (36.9)
Male	309 (66.2)	270 (67.2)	39 (60.0)
Transgender	13 (2.8)	11 (2.7)	2 (3.1)
Race/ethnicity			
African–American/Black	209 (44.5)	188 (46.4)	21 (32.3)
American Indian/American Native	14 (3.0)	14 (3.0)	0 (0.0)
Asian American/Pacific Islander	11 (2.3)	9 (2.2)	2 (3.1)
Hispanic/Latinx	42 (8.9)	33 (7.0)	9 (13.8)
NH White	135 (28.7)	115 (28.4)	20 (30.8)
Other/more than one ethnic group	57 (12.1)	44 (10.9)	13 (20.0)
Cigarettes smoked per day—mean (SD)	11.3 (10.5)	11.0 (10.4)	13.0 (11.2)
First cigarette after waking			
Within 5 min	171 (36.4)	50 (31.1)	29 (44.6)
6–30 min	98 (20.9)	35 (21.7)	18 (27.7)
31–60 min	74 (15.7)	25 (15.5)	5 (7.7)
After 60 min	126 (26.9)	51 (31.7)	13 (20.0)
Past-year intentional quit attempt lasting 1 day or more	208 (44.3)	175 (43.2)	33 (50.8)
Source of e-cigarettes			
Smoke/vape shop or marijuana dispensary	--	--	35 (53.8)
Friends/strangers	--	--	31 (47.7)
Liquor/convenience store or gas station	--	--	12 (18.5)
Supermarket or drug store	--	--	2 (5.7)

Note. Percentages do not total 100; missing data on gender, ethnicity and first cigarette after waking (≤1.0%).

**Table 2:** Patterns of e-cigarette use and association with cigarette quit attempts among dual users (*n* = 65).

Variable	Quit Attempt in the Past Year	
	No Quit Attempt (*n* = 32)	Quit Attempt (*n* = 32)
	*n* (%)	*n* (%)
Times per day e-cigarette is used ^1^—mean (SD)	4.8 (6.4)	6.4 (7.4)
Duration that e-cigarette was used each day		
Just a few puffs (<1 min)	14 (43.8)	10 (31.3)
1–5 min	6 (18.8)	7 (21.9)
6–15 min	6 (18.8)	5 (15.6)
16–60 min	3 (9.3)	6 (18.8)
>1 h	3 (9.3)	4 (12.5)
How e-cigarette is used		
Throughout day, continuously	8 (25.0)	11 (34.4)
Bouts shorter than smoking a cigarette	18 (56.3)	14 (43.8)
Bouts similar to smoking a cigarette	4 (12.5)	6 (18.8)
Other	2 (6.3)	1 (3.1)
Knowledge of nicotine concentration		
Don’t know	21 (65.6)	16 (50.0)
0 mg	0 (0.0)	0 (0.0)
1–6 mg	2 (6.3)	7 (21.9)
7–12 mg	3 (9.4)	2 (6.3)
13–18 mg	1 (3.1)	4 (12.5)
19–24 mg	0 (0.0)	1 (3.1)
25+ mg	5 (15.6)	2 (6.3)

Note. Missing data on quit attempt from one dual user (1.5%). ^1^ One time is 15 puffs or using an e-cigarette for 10 min.

**Table 3:** Attitudes toward cigarettes and e-cigarettes among cigarette-only smokers and dual users reporting agree or strongly agree.

Variable	Cigarette-Only (*n* = 405)	Dual Users (*n* = 65)
	*n* (%)	*n* (%)
Cigarette smoking cessation		
People who smoke should quit	299 (74.0)	46 (70.8)
E-cigarettes help people quit using cigarettes	190 (47.2)	33 (50.8)
Use of tobacco or e-cigarettes		
Cigarettes should be allowed outdoors	239 (59.3)	39 (60.0)
E-cigarettes should be allowed outdoors	280 (69.3)	47 (72.3)
Cigarettes should be allowed indoors	59 (14.6)	8 (12.3)
E-cigarettes should be allowed indoors	137 (33.9)	27 (41.5)
Perceptions of cigarette or e-cigarette harm and safety		
Cigarette smoke is dangerous to babies/kids	387 (96.0)	61 (93.9)
E-cigarette vapor is dangerous to babies/kids	319 (79.2)	53 (81.5)
E-cigarettes are safer than smoking	146 (36.1)	32 (49.2)
Nicotine and tar content		
E-cigarettes don’t contain tar	94 (23.3)	15 (23.1)
Cigars/cigarillos have less nicotine than cigarettes	96 (23.8)	14 (21.5)
Cigars/cigarillos have less nicotine than e-cigarettes	106 (26.4)	15 (23.1)

**Table 4:** Relationships between quit attempts and demographic characteristics, tobacco use patterns and attitudes toward smoking cigarettes and using e-cigarettes.

	Quit Attempt in the Past Year	
	Bivariate OR (95% CI)	Multivariate aOR (95% CI)
Age	1.01 (0.99, 1.02)	1.01 (0.99, 1.03)
Gender (female referent)		
Male/transgender	0.88 (0.59, 1.30)	0.76 (0.45, 1.31)
Race/ethnicity (NH White referent)		
Non-White	1.01 (0.99, 1.02)	1.01 (0.99, 1.02)
Cigarettes smoked per day	0.96 (0.94, 0.99)	0.98 (0.95, 1.01)
First cigarette within 30 min of waking ^1^	0.48 (0.33, 0.70)	0.57 (0.33, 0.96)
Current e-cigarette use ^2^	1.32 (0.76, 2.30)	1.59 (0.87, 2.87)
Attitudes toward e-cigarette use		
People who smoke should quit	2.21 (1.43, 3.42)	2.15 (1.22, 3.79)
E-cigarettes help people quit using cigarettes	1.36 (0.94, 1.97)	1.58 (0.91, 2.75)
E-cigarettes are safer than smoking	0.96 (0.66, 1.40)	0.72 (0.41, 1.28)
E-cigarettes don’t contain tar	0.74 (0.47, 1.14)	0.82 (0.45, 1.49)

Note. OR = odds ratio. aOR = adjusted odds ratio. NH = non-Hispanic. ^1^ Smoking first cigarette within 30 min after waking up in the morning. ^2^ Use of an e-cigarette in the past 30 days.
